# Temporal inverted internal limiting membrane flap technique for a 500 μm full-thickness macular hole: A case report

**DOI:** 10.1097/MD.0000000000051227

**Published:** 2026-10-23

**Authors:** Sebastian Sirek, Justyna Sierocka-Stępień, Mikołaj Guzikowski, Sabina Kowalczyk, Dawid Woszczek, Piotr Dudek, Dorota Wyględowska-Promieńska

**Affiliations:** a Department of Ophthalmology, Faculty of Medical Sciences in Katowice, Medical University of Silesia, Katowice, Poland; b Department of Ophthalmology, Prof. K. Gibinski University Clinical Center, Medical University of Silesia in Katowice, Katowice, Poland; c Department of Ophthalmology, Students’ Scientific Club, Faculty of Medical Sciences in Katowice, Medical University of Silesia, Katowice, Poland.

**Keywords:** case report, full-thickness macular hole, pars plana vitrectomy, temporal inverted flap, vitreoretinal surgery

## Abstract

**Rationale:**

Large full-thickness macular holes (FTMH), defined by the International Vitreomacular Traction Study Group as defects of all retinal layers within the foveal region with an aperture size of 400 μm or more, remain a challenging condition with reduced anatomical closure rates using conventional techniques. We report a case managed with the temporal inverted internal limiting membrane (ILM) flap technique.

**Patient concerns:**

A 70-year-old female patient presented to the Outpatient Ophthalmology Clinic with decreased vision in the left eye.

**Diagnoses:**

Decimal Snellen best-corrected visual acuity was 0.3 (5/16) in oculus sinister (left eye) and 0.06 (3/50) in oculus dexter (right eye), and intraocular pressure was 16 mm Hg in both eyes. Optical coherence tomography (OCT) revealed an hourglass-shaped FTMH with cystic changes within the retina and rounded thickened edges, with a minimum aperture size of 500 μm. Fundus examination and OCT of the fellow (right) eye also revealed a full-thickness macular hole (MH), with a smaller aperture than the left eye.

**Interventions:**

The patient underwent 25G pars plana vitrectomy with the temporal inverted ILM flap technique, followed by fluid–air exchange and 25% sulfur hexafluoride gas endotamponade.

**Outcomes:**

Follow-up OCT at 33 and 45 days after the procedure demonstrated complete closure of the MH, with a small residual fluid pocket beneath the inverted ILM flap noted at day 33. Best-corrected visual acuity was 0.1 (5/50) in oculus dexter (right eye) and 0.5 (5/10) in oculus sinister, and intraocular pressure was 15 mm Hg in both eyes at the final follow-up.

**Lessons:**

In the presented patient, 25G vitrectomy using the temporal inverted ILM flap technique resulted in short-term anatomical closure of the MH and improvement in visual acuity, consistent with previous reports of this technique in the management of large FTMH.

## 1. Introduction

Large full-thickness macular holes (FTMH), according to the definition of the International Vitreomacular Traction Study Group, are defects of all retinal layers located within the foveal region, with an aperture size of 400 μm or more.^[1]^ In such large macular holes (MH), the use of standard pars plana vitrectomy with internal limiting membrane (ILM) peeling and gas endotamponade results in a closure rate of 80%. This outcome, compared with almost 100% closure rates in small and medium MHs according to the International Vitreomacular Traction Study classification, is unsatisfactory.^[2]^ To manage such large MHs, new surgical techniques have been developed, including the inverted flap technique introduced by Michalewska Z et al,^[3]^ and its modification, the temporal inverted flap, described by Michalewska Z et al.^[4]^

A new CLOSE classification has been proposed, which divides large MHs into subgroups depending on the surgical technique providing the best results in a given size group.^[5]^

A cutoff point at the level of 650 μm has also been observed, which represents a threshold of increased risk of unsuccessful surgery with the standard technique and a strong indication for the use of new surgical techniques for large MHs.^[6]^

Although conventional ILM peeling achieves high closure rates up to approximately 650 μm, the temporal inverted flap technique was selected in this case despite the 500 μm aperture because of the 6-month duration of symptoms, the relatively wide 980 μm base, the presence of thickened cystic edges, and the concurrent epiretinal membrane, which were judged to increase the risk of unsuccessful closure with peeling alone. It should be noted that recent evidence is not uniformly favorable to this choice: Chen et al reported poorer visual and external limiting membrane recovery with the inverted flap compared with conventional ILM peeling in idiopathic holes <650 μm.^[7]^ This does not invalidate the outcome of the present case but is relevant to the rationale for technique selection in holes of this size.

The aim of this case report is to describe the technique and short-term clinical and anatomical outcome of the temporal inverted ILM flap in a patient with a large MH.

## 2. Methods

A 70-year-old female patient presented to the Outpatient Ophthalmology Clinic due to progressive deterioration of vision in the left eye that had been present for approximately 6 months. The patient also reported metamorphopsia in the form of image distortion affecting the left eye. The MH was considered idiopathic. No history of ocular trauma, relevant medication exposure, or family history of macular disease was identified.

Her ocular history included bilateral pseudophakia. The patient had no history of systemic chronic diseases. Manifest refraction was +0.75 D sphere, −0.50 D cylinder at 93° in the right eye and +0.75 D sphere, −0.50 D cylinder at 99° in the left eye. No history of high myopia was documented, axial length measurements were unavailable. At admission, best-corrected visual acuity (BCVA) was 0.06 (3/50) in oculus dexter (right eye) (OD) and 0.3 (5/16) in oculus sinister (left eye) (OS), and intraocular pressure (IOP) was 16 mm Hg in both eyes. BCVA was recorded using decimal Snellen visual acuity notation, with Snellen fraction equivalents provided in parentheses.

Optical coherence tomography (OCT) revealed an hourglass-shaped FTMH with cystic changes within the retina and rounded thickened margins. A complete loss of retinal layers from the ILM to the retinal pigment epithelium was visible. OCT measurements of the MH showed a minimum aperture size of 500 μm, a base diameter of 980 μm, and a height of 520 μm. The left arm length of the MH was 440 μm and the right arm length was 430 μm. (Fig. 1). Preoperative OCT also demonstrated the presence of an epiretinal membrane. Fundus examination and OCT of the fellow (right) eye similarly revealed a full-thickness MH; however, its aperture was smaller than that observed in the left eye. Given the greater aperture size of the left-eye MH, surgical intervention was prioritized for that eye, while the right-eye MH, despite the lower measured BCVA, was managed conservatively with continued clinical observation given its smaller aperture. The minimum aperture diameter of the right-eye MH was not formally measured, though it was visibly smaller than that of the left eye on OCT; the duration of symptoms in the right eye was unknown. This comparison was therefore qualitative rather than a formally quantified risk assessment, no additional documented factors such as patient preference, estimated visual potential, or a staged bilateral treatment plan influenced the decision to prioritize the left eye. The patient underwent 25-gauge pars plana vitrectomy using the temporal inverted flap technique.

**Figure 1.**
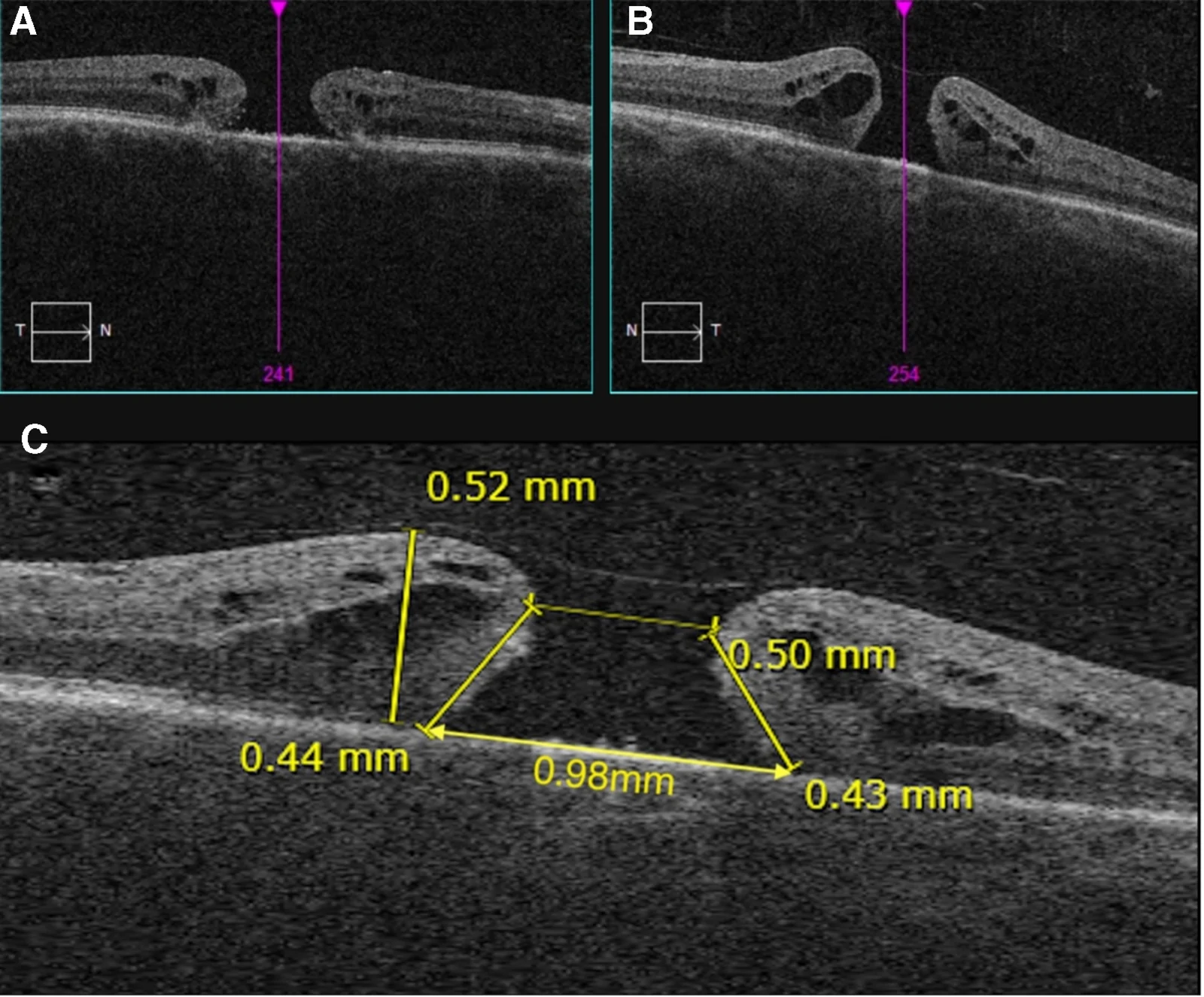
OCT examination performed with a Carl Zeiss Meditec device. (A) OCT of the right eye, showing the smaller full-thickness macular hole. (B) OCT of the left eye, showing the larger full-thickness macular hole. (C) OCT of the left eye with FTMH measurements. FTMH = full-thickness macular holes, OCT = optical coherence tomography.

Anesthesia was achieved with a sub-tenon injection of 2% lidocaine and 0.5% bupivacaine solution in a 1:1 ratio, administered in the inferonasal quadrant. Three trocars were placed at 5, 2, and 10 o’clock positions. Infusion was connected in the inferotemporal quadrant. A core vitrectomy was performed, followed by surgical induction of posterior vitreous detachment. The epiretinal membrane identified on preoperative OCT was removed with microforceps prior to ILM staining and peeling. Subsequently, ILM within the vascular arcade was stained with Brilliant Blue G, applied for approximately 20 seconds before being washed out with balanced salt solution, to improve its visualization (Fig. 2A and B). Using circular movements while simultaneously holding the edge of the external crescent-shaped membrane with microforceps, ILM peeling was performed. The flap, measuring approximately 1.5 optic disc diameters, remained attached at its temporal base and was inverted nasally, with its free edge tucked into the MH and positioned to cover its surface (Fig. 2C–F). Fluid to air exchange was performed, followed by air to 25% sulfur hexafluoride exchange, which represented the final intraocular tamponade concentration. The patient was advised to maintain a face-down position for 2 weeks postoperatively. This positioning duration was intended to promote flap stability and apposition against the retinal pigment epithelium during the early phase of anatomical closure. Adherence to and tolerability of this positioning regimen were not formally assessed or documented.

**Figure 2.**
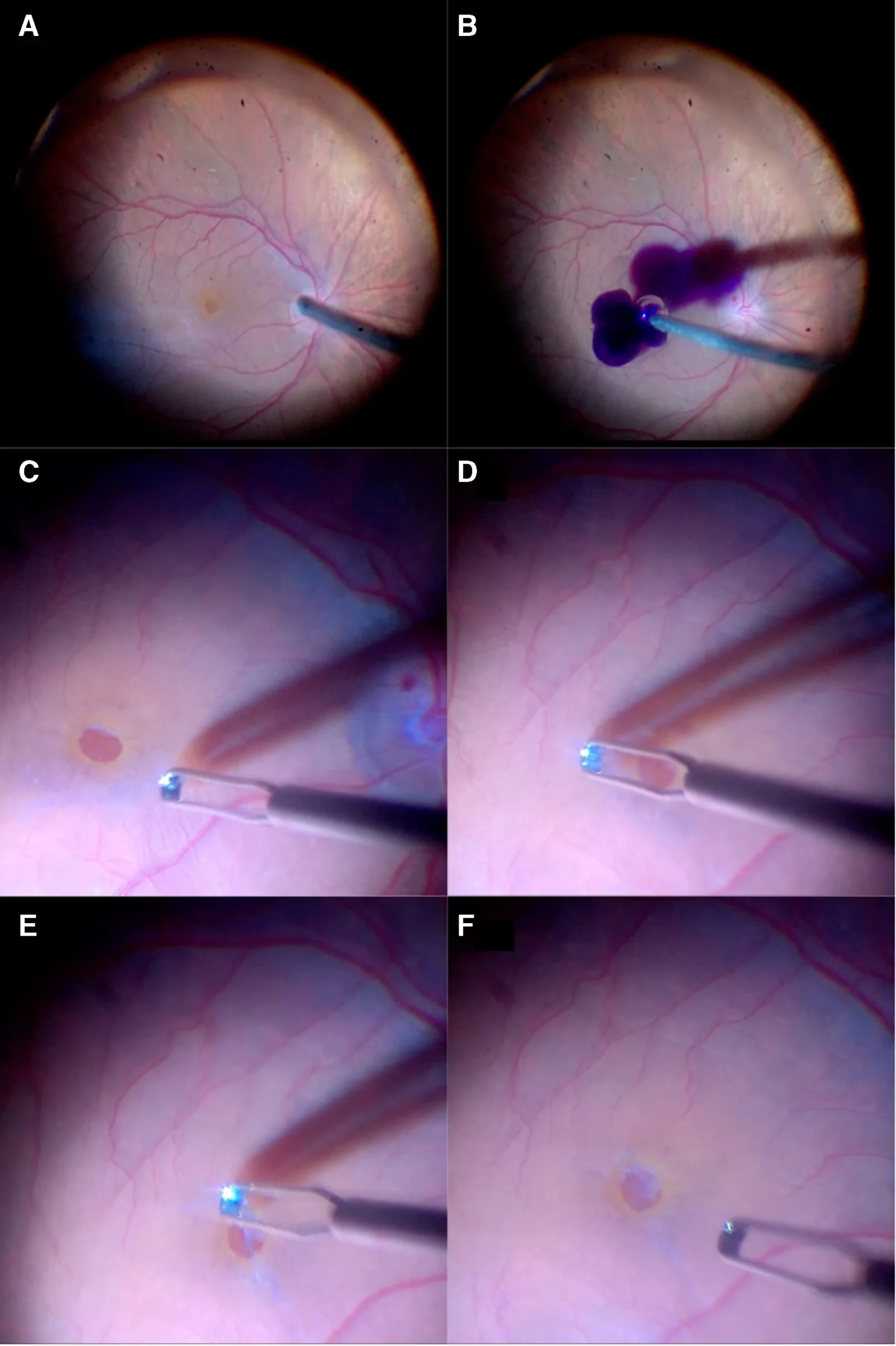
Intraoperative sequence of the inverted ILM flap technique. (A) Initial intraoperative view of the full-thickness macular hole before membrane staining. (B) Staining of the internal limiting membrane with Brilliant Blue G to enhance its visualization. (C) Initiation of ILM peeling around the macular hole using microforceps. (D) Continuation of circumferential ILM peeling while preserving a remnant attached to the margin of the macular hole. (E) Placement of the temporally based, nasally inverted ILM flap (approximately 1.5 optic disc diameters), with its free edge tucked into the macular hole and positioned to cover its surface. (F) Final intraoperative appearance showing the ILM flap properly positioned within the macular hole before completion of surgery. ILM = internal limiting membrane.

Ethics committee approval was not required because this study is a single-patient case report describing routine clinical care, consisting of a descriptive analysis without any intervention beyond standard treatment. Written informed consent was obtained from the patient for the publication of this case report and accompanying clinical images. The report was prepared using anonymized patient data in accordance with applicable ethical standards and institutional practice.

## 3. Results

The procedure was performed in a standard manner without complications. One day after surgery, BCVA was 0.06 (3/50) in OD and hand movements in OS; the markedly reduced visual acuity in the operated eye was attributable to the presence of intraocular gas tamponade in the immediate postoperative period. IOP was 15 mm Hg in both eyes.

In the follow-up OCT examination performed 33 days after surgery, complete closure of the MH was observed, with a small residual fluid pocket beneath the inverted ILM flap; the surrounding foveal architecture was otherwise preserved (Fig. 3). BCVA was 0.2 (5/25) in OD and 0.3 (5/16) in OS, while IOP was 18 mm Hg in OD and 21 mm Hg in OS. The IOP of 21 mm Hg in the operated eye at day 33 was an isolated observation that did not require intervention and normalized without treatment by day 45. At the subsequent follow-up 45 days after surgery, BCVA was 0.1 (5/50) in OD and 0.5 (5/10) in OS, and IOP was 15 mm Hg in both eyes. A summary of key clinical time points, from baseline through postoperative day 45, is provided in Table 1. OCT was also performed, which revealed complete closure of the MH with resolution of the previously noted subflap fluid pocket (Fig. 4). The fluctuation in right-eye BCVA over follow-up (0.06 at baseline, 0.2 at day 33, and 0.1 at day 45) occurred in the absence of any surgical intervention and represents observed variability in an untreated eye rather than a documented change in the right-eye MH; the same refraction and visual acuity testing protocol was used at each visit. No clinically apparent anatomic change was observed on right-eye OCT during the same period.

**Table 1:** Clinical timeline.

Time point	Event
Baseline	BCVA 0.06 (OD)/0.3 (OS); IOP 16 mm Hg both eyes; OCT: FTMH 500 μm (OS), smaller FTMH (OD)
Day 0	25G PPV with temporal inverted ILM flap, OS
Day 1	BCVA 0.06 (OD)/HM (OS); IOP 15 mm Hg both eyes
Day 33	BCVA 0.2 (OD)/0.3 (OS); IOP 18 mm Hg (OD)/21 mm Hg (OS); OCT: MH closure, residual subflap fluid
Day 45	BCVA 0.1 (OD)/0.5 (OS); IOP 15 mm Hg both eyes; OCT: complete closure, fluid resolved

BCVA = best-corrected visual acuity, FTMH = full-thickness macular hole, HM = hand movements, ILM = internal limiting membrane, IOP = intraocular pressure, MH = macular hole, OCT = optical coherence tomography, OD = oculus dexter (right eye), OS = oculus sinister (left eye), PPV = pars plana vitrectomy.

**Figure 3.**
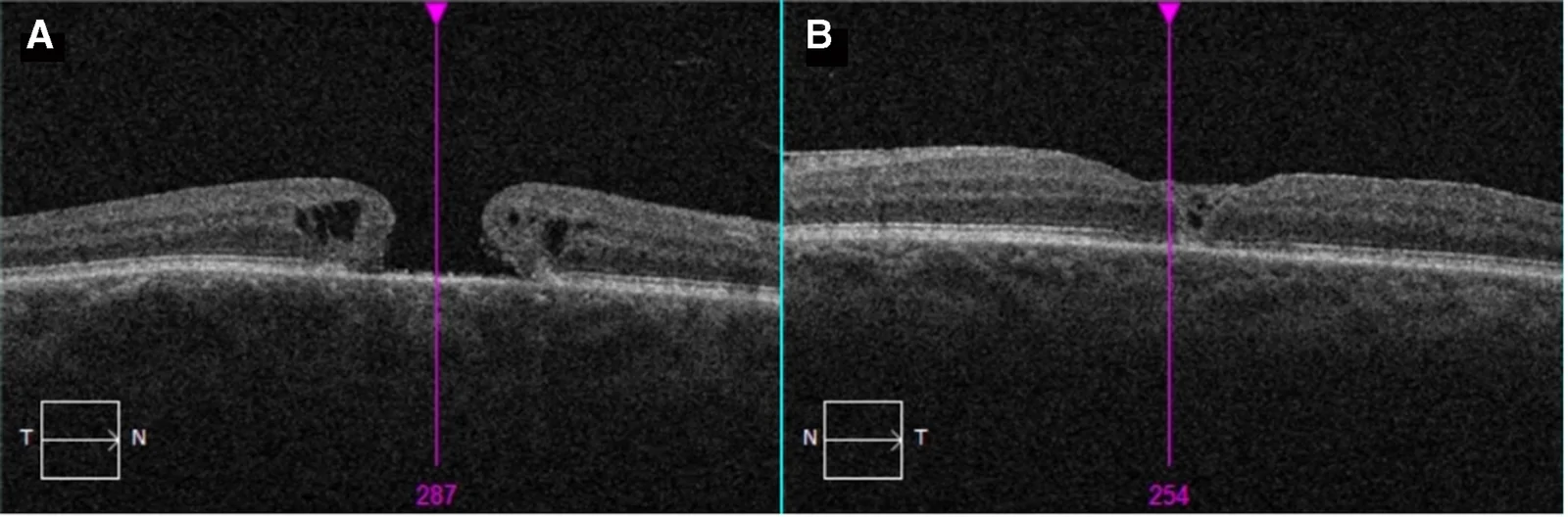
Preoperative and postoperative OCT findings after inverted ILM flap surgery. (A) Preoperative OCT image demonstrating a full-thickness macular hole with disruption of the foveal architecture. (B) OCT image obtained 33 days after surgery showing complete anatomical closure of the macular hole with residual foveal/outer-retinal irregularity. A small residual fluid pocket is visible beneath the inverted ILM flap; however, the surrounding foveal architecture remains preserved. ILM = internal limiting membrane, OCT = optical coherence tomography.

**Figure 4.**
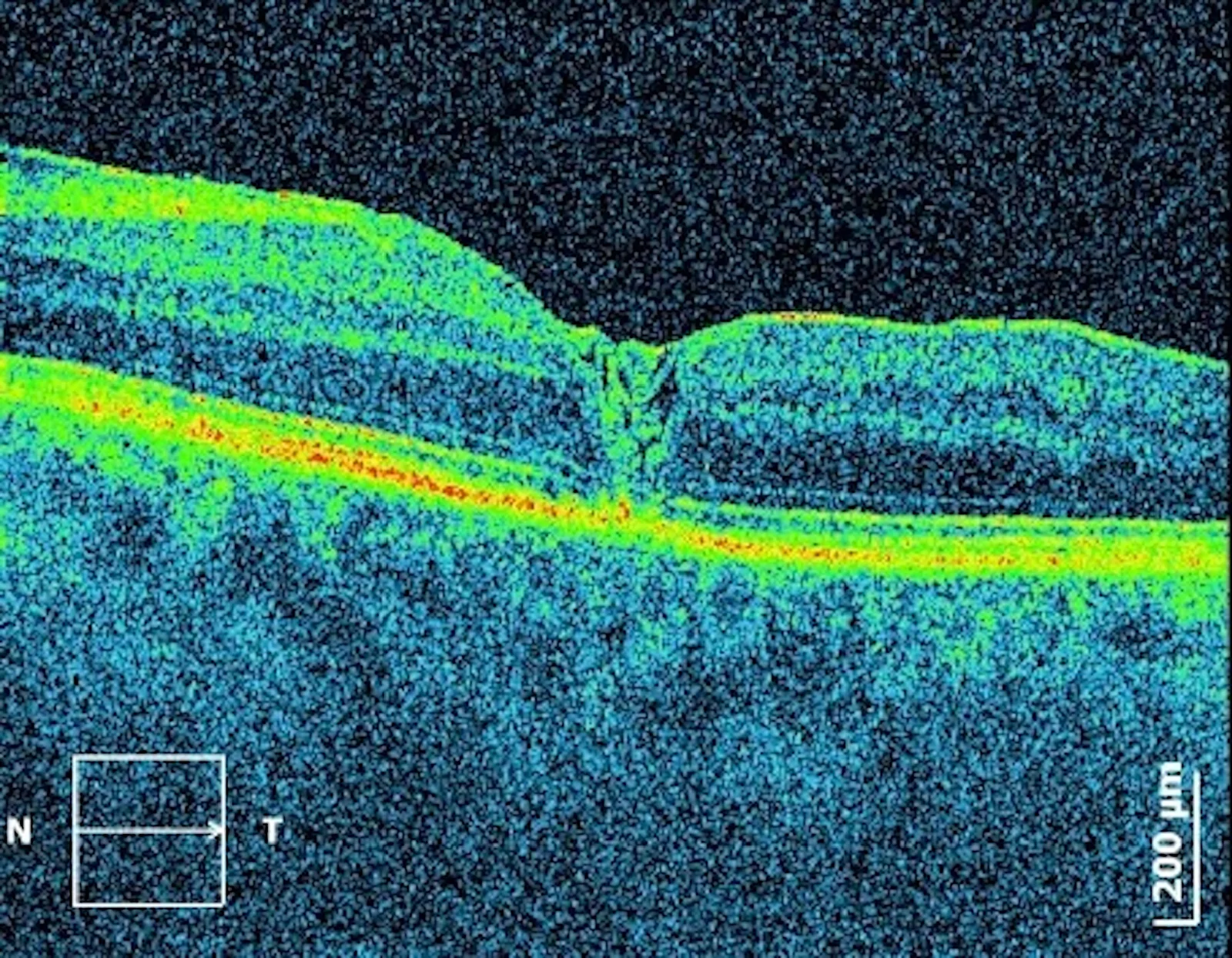
Postoperative OCT findings 45 days after inverted ILM flap surgery. The OCT image demonstrates anatomic closure of the macular hole with residual foveal/outer-retinal irregularity. The previously observed small residual fluid pocket beneath the inverted ILM flap has completely resolved, with preserved surrounding retinal architecture. This image was acquired using a different OCT acquisition setting than that used in Figure 3, which accounts for the difference in image appearance; both images were obtained using the same Carl Zeiss Meditec device. ILM = internal limiting membrane, OCT = optical coherence tomography.

## 4. Discussion

Two main techniques using an inverted flap of the ILM are commonly applied in the treatment of large MHs: the classic inverted flap method and its modified version, the temporal inverted flap.

Michalewska et al,^[4]^ who introduced both approaches, reported that the success rates for MH closure and visual acuity improvement were comparable between the 2 techniques. Because the outcomes were similar, the authors concluded that the ILM flap does not need to be particularly large, supporting the practicality of the temporal inverted flap. Moreover, this approach was associated with a lower incidence of nerve fiber layer splitting.^[4]^

Elgouhary et al^[8]^ directly compared the temporal inverted flap technique with a multilayered inverted flap technique (created from several ILM layers, inverted and placed over the MH), rather than with the classic inverted flap. Closure patterns were 60% U-shaped, 10% V-shaped, 20% W-shaped, and 10% unclosed in the temporal flap group, versus 70% U-shaped, 20% V-shaped, and 10% W-shaped in the multilayered flap group. U-shaped closure correlated with better postoperative visual acuity in both groups.^[8]^ Comparisons among different flap configurations (temporal, classic, and multilayered) should be distinguished from comparisons of any flap technique against conventional ILM peeling, as reported outcomes may also depend on the diameter of the treated hole. Collectively, these studies indicate that the temporal inverted ILM flap technique is already well established for the management of large FTMH. Accordingly, the value of the present report lies not in demonstrating a novel technique, but in providing a detailed technical illustration, perioperative OCT documentation, and short-term clinical course in an individual case with a 500 μm MH.

Recovery of the external limiting membrane (ELM) was also evaluated. Elgouhary et al^[8]^ observed ELM restoration in 40% of cases after the temporal flap and 50% after the multilayered inverted flap, 6 months after surgery, with improvements in visual acuity in both groups.^[8]^ Another study reported ELM restoration in 53% of cases after 1 month and 79% after 3 months, also correlating with visual improvement.^[9]^

Hirata et al^[10]^ highlighted a limitation of the temporal inverted flap: in younger patients, or when the ILM flap was particularly large, flap contraction could occur, leading to reduced postoperative visual acuity.^[10]^

The transient subflap fluid pocket observed at day 33 in this case resolved spontaneously by day 45 without compromising anatomical closure. This finding may serve as a practical observation for clinicians; however, given the single-case nature of this report, it should be interpreted descriptively and should not be taken to imply that such resolution is an expected pattern across all cases.

Teng and Zhang^[11]^ also applied the temporal inverted flap in patients with MH retinal detachment and followed them for 12 months. They achieved MH closure and retinal reattachment in 100% of cases. BCVA improved, and macular anatomy was restored in 94.4% of patients. According to Teng and Zhang, combining vitrectomy with the temporal inverted flap technique represented an effective approach to restoring foveal architecture in their cohort.^[11]^

A comparative study by Fathy et al^[12]^ focused on large FTMHs. In the temporal inverted flap group, closure was achieved in 100% of cases, while the ILM peeling group achieved 85%. Restoration of a U-shaped fovea – closest to normal anatomical conditions – was more common with the temporal inverted flap. During follow-up, the proportion of U-shaped foveae increased progressively: 85% at 1 month, 95% at 3 months, and 100% at 6 months. In contrast, the ILM peeling group remained at 55% across all follow-ups.^[12]^

Avci et al^[13]^ reported similar results. In their study, MH closure was achieved in 100% of eyes using the temporal inverted flap and in 72.2% of those treated with ILM peeling. U-shaped foveal architecture was restored in 80% of temporal flap cases but in only 5.5% of eyes after ILM peeling.^[13]^

The presence of bilateral full-thickness MHs in this patient is also clinically noteworthy. Surgical treatment was prioritized in the eye with the larger MH rather than in the eye with the worse baseline BCVA. The surgeon judged the qualitatively larger aperture to represent the greater surgical priority, although this assessment was not based on a formally quantified risk comparison.

## 5. Limitations

This report has some limitations, including the relatively short postoperative follow-up period of 45 days. However, the primary aim of this case report was to illustrate the application and short-term outcome of the inverted ILM flap technique in the treatment of a large full-thickness MH. Within the available follow-up period, optical coherence tomography demonstrated complete MH closure with resolution of the previously observed subflap fluid pocket and preservation of the surrounding retinal architecture. Longer-term follow-up would be valuable to further assess the stability of these anatomical findings and the evolution of functional outcomes. Biometric data were not measured due to the lack of need for phacoemulsification in this pseudophakic patient, which is a limitation given the incomplete picture of the anatomical conditions.

## 6. Conclusion

In the presented patient, 25G vitrectomy with the temporal inverted ILM flap technique^[4]^ resulted in anatomical closure of a 500 μm MH and improvement in visual acuity during 45 days of follow-up. This case illustrates the temporal inverted ILM flap as a technically feasible option associated with short-term anatomical closure and visual improvement in a large full-thickness MH, without implying comparative superiority over conventional ILM peeling or establishing a specific size threshold for technique selection.

## Acknowledgments

During the preparation of this manuscript, the authors used ChatGPT (OpenAI) to assist with translation of the text into English. The authors have reviewed and edited the output and take full responsibility for the content of this publication.

## Author contributions

**Conceptualization:** Sebastian Sirek, Dorota Wyględowska-Promieńska.

**Investigation:** Mikołaj Guzikowski, Sabina Kowalczyk, Dawid Woszczek, Piotr Dudek.

**Methodology:** Sebastian Sirek.

**Project administration:** Sebastian Sirek.

**Software:** Justyna Sierocka-Stępień, Piotr Dudek.

**Supervision:** Dorota Wyględowska-Promieńska.

**Visualization:** Justyna Sierocka-Stępień, Mikołaj Guzikowski, Sabina Kowalczyk, Dawid Woszczek.

**Writing – original draft:** Mikołaj Guzikowski, Sabina Kowalczyk, Dawid Woszczek.

**Writing – review & editing:** Sebastian Sirek, Dorota Wyględowska-Promieńska.
